# Endotoxin Detection in Magnetic Resonance Imaging Contrast Agent Using Optimising Chromogenic Limulus Amebocyte Lysate Assay

**DOI:** 10.21315/mjms2024.31.5.20

**Published:** 2024-10-08

**Authors:** Atiqah Ab Aziz, Sahrinanah Mappiare, Hui Yin Nam, Durga Devi, Mohd Rafie Johan, Tunku Kamarul

**Affiliations:** 1 Tissue Engineering Group (TEG), National Orthopaedic Centre of Excellence for Research and Learning (NOCERAL), Department of Orthopaedic Surgery, Faculty of Medicine, Universiti Malaya, Kuala Lumpur, Malaysia; 2 M. Kandiah Faculty of Medicine and Health Sciences, Universiti Tunku Abdul Rahman, Selangor, Malaysia; 3 Nanotechnology and Catalysis Research Center (NANOCAT), Institute for Advanced Studies, Universiti Malaya, Kuala Lumpur, Malaysia

**Keywords:** Limulus amebocyte lysate, contrast media, chromogenic, endotoxin, pyrogen, horseshoe crab, medical devices, bacterial

## Abstract

Endotoxin contamination in magnetic resonance imaging (MRI) contrast agents can pose a risk to patient safety causing immune reactions. Strict endotoxin limits are enforced for implants and catheters inserted into the body, but there are not clear rules for MRI contrast agents. Here, we investigated the efficacy of chromogenic LAL assay test for screening endotoxin activity in MRI contrast media manufactured in Malaysia. The powdered agent was dissolved in water for injection and endotoxin levels were measured. The coefficient of efficiency value for the standard curve, exhibiting *r*^2^ ≥ 0.98, along with the absence of interfering substances and endotoxin activity below the regulatory threshold of 0.5 EU/mL, support the conclusion that the agent is unlikely to be pyrogenic or induce pyrogenic effect.

## Introduction

Magnetic resonance imaging (MRI) contrast agents enhance the distinction between normal and abnormal tissues and improve the visualisation of specific structures or pathologies (1). These agents can potentially become contaminated during the manufacturing process or because of improper handling or storage (2). While sterility during the production of contrast agents ensures the absence of viable microorganisms, it does not guarantee that they are free from pyrogens. Pyrogen-free status refers to the absence of substances that can cause a pyrogenic response in the body, such as endotoxins (3). Both sterility and pyrogen-free status are crucial for ensuring patient safety.

To ensure that a contrast agent is pyrogen-free, additional tests are conducted specifically for endotoxins. Endotoxins are natural compounds found in the outer cell membrane of Gram-negative bacteria and can lead to cell death by triggering complement activation (4). This immune response can result in fever, chills, allergic reactions or other serious complications (5). The *Limulus* amebocyte lysate (LAL) assay, which employs a component of horseshoe crab (*Limulus polyphemus*) blood called amoebocytes, is the most widely recommended method by regulatory bodies (6, 7). The decision of using chromogenic LAL method is primarily attributed to its exceptional sensitivity and specificity. This assay demonstrates a remarkable ability to detect even the smallest amount of endotoxins, allowing for accurate detection at levels relevant to patient safety (8).

Meeting both sterility and pyrogen-free requirements for contrast agents is essential to minimise the risk of adverse reactions when these agents are administered to patients (9). The contrast agent used in this study is a proprietary synthetic material composed of iron oxide nanoparticles in a dry powder form. It is intended to be administered to humans via small volume parenteral (SVP) preparation. It is manufactured either as a powder or as an aqueous colloidal injection solution in 5% dextrose. Both forms require sterilisation and are administered intravenously as MRI contrast agents. The SVP administration method necessitates the assessment of pyrogenicity safety. This study aimed to evaluate endotoxin contamination in sterile contrast agents available in Malaysia.

## Methods

### Sample

The contrast agent was obtained from one manufacturer in Malaysia and tested for endotoxins. The bottle of contrast agent was taken randomly from the same lot.

### Selection and Preparation of Sample

A sample of the contrast agent powder was taken using a sterile spatula, weighed and placed in a borosilicate bottle. The entire process took place within a Class II biosafety cabinet to ensure a controlled environment. All glassware employed underwent depyrogenation at 200 °C prior to use. The contrast agent was prepared at a concentration of 0.2 g/mL, utilising water for injection (Infusol) as the polar vehicle.

### Sample Extraction Method

The reconstituted contrast agent was agitated using a shaking incubator for 72 h at 160 rpm and 37 °C. The solution was utilised for a maximum of 24 h after extraction to prevent sorption onto the extraction container or any alterations in composition. The solution was filtered via a non-pyrogenic syringe (10 mL) equipped with a non-pyrogenic filter membrane to eliminate suspended particulates. The pH of the solution was adjusted to 6–8 using endotoxin-free 0.1 M NaOH or 0.1 M HCl. A minimal quantity was extracted from the complete sample to mitigate the risk of pH electrode contamination. The remaining sample was stored at 2 °C–8 °C for less than 24 h and at −20 °C for periods surpassing 24 h, effectively inhibiting all bacteriological activity. For the subsequent step, the contrast agent solution in the borosilicate bottle was transferred to the biosafety cabinet and subsequently pipetted into a 96-well plate.

### Endotoxin Assay

This study employed the chromogenic technique in accordance with the International Pharmacopoeia (7) and ISO 10993-11 (10). The updated LAL chromogenic test was conducted using the Pierce Chromogenic Endotoxin Quant Kit (Cat. No. A39552) (Thermo Fisher Scientific). This kit is compatible with β-glucans.

### Preparation of Endotoxin Standard Solution

The endotoxin standard was reconstituted with endotoxin-free water at room temperature by adding 1/10 mL of the specified EU quantity to the *E. coli* endotoxin standard vial to make 10 EU/mL endotoxin stock solution. The solution was vortexed for 15 min at 1,500 rpm. The reconstituted stock solution remained stable for 4 weeks when stored at 2 °C–8 °C. Prior to use, the solution was allowed to reach room temperature and vigorously mixed for 15 min.

### Preparation of Interfering Substances and Sample Solutions

The presence of interfering substances in test samples can potentially result in product inhibition, leading to false negative results. Therefore, it is recommended to assess potential product inhibition for each sample type, whether undiluted or suitably diluted (e.g. with serum). To confirm the presence of inhibition, the sample was added with 0.1 EU/mL endotoxin. Both spiked and unspiked solutions were created through 2-fold dilutions. The diluted spiked test item was analysed in conjunction with the unspiked test item dilutions. The discrepancy in absorbance between unspiked and spiked test item dilutions should fall within the range of spiked ± 25%.

### Assay Procedure

The microplate reader was prepared at 405 nm, and the microplate reader software was set for the 96-well plate layout. The plate was pre-equilibrated in a heating block at 37 ± 1 °C for 10 min. Maintaining the microplate at 37 ± 1 °C, 50 mu;L of each standard, sample and interfering substances were added to their respective microplate wells. The procedure and measurement principles were carried out according to the manufacturer’s protocol (11). Briefly, the intensity of yellow chromogen released following synthetic substrate cleavage was measured and quantified.

## Results

**Figure 1:**
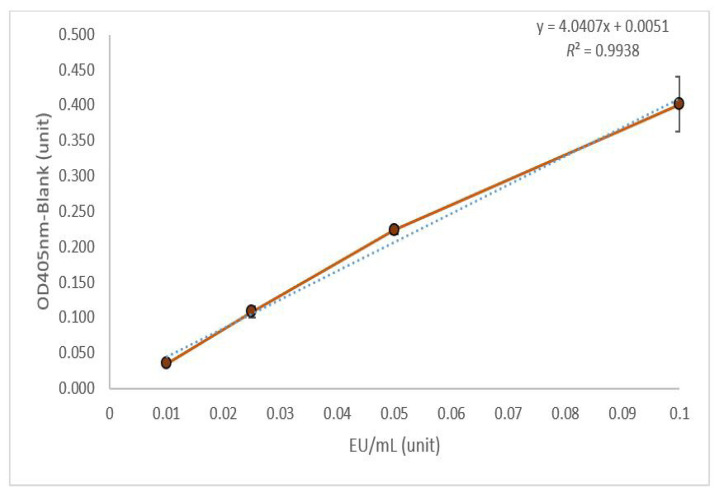
Standard curve for the low standard endotoxin concentration

The optical densities of the blank, standard solutions, interfering substances, and samples are presented in Table 1. These data represent the raw optical density (OD) values obtained from the microplate reader. The standard curve for low standard endotoxin concentration is depicted in Figure 1.

The findings summarised in Table 1 and Figure 1 reveal that the endotoxin concentration of the sample was within or close to the range of the endotoxin solution concentration. Additionally, the results in Table 2 provide a comparison between the unspiked and spiked sample, indicating either the absence or presence of interfering substances based on the difference value being above or below the spiked known endotoxin amount (acceptable range for interfering substances: 0.005 EU/mL–0.095 EU/mL).

Consequently, considering the standard solution has a linear curve with *r*^2^ ≥ 0.98, the absence of interfering substances and the endotoxin activity of the test item was below the regulatory limit of 0.5 EU/mL, it was concluded that the test item was unlikely to possess pyrogenic properties or induce pyrogenic effects.

## Discussion

MRI contrast agents are generally stable. However, it can undergo oxidation under certain conditions, such as exposure to reactive oxygen species (ROS) like hydrogen peroxide or free radicals (12). These species can interact with the contrast agent and cause oxidation. Some contrast agents may inherently possess a degree of chemical instability, making them more prone to oxidation reactions. Moreover, contrast agents can be sensitive to environmental factors such as temperature, light and humidity (13). Improper storage conditions, such as exposure to high temperatures or prolonged storage times, can promote oxidation. Oxidation of MRI contrast agents can lead to changes in their properties, including altered pH, decreased stability or potential toxicity. Therefore, it is crucial to handle and store these agents properly to minimise the risk of oxidation.

The LAL test is a highly sensitive assay. In the LAL test, a sample suspected of containing endotoxins is mixed with a reagent derived from the blood cells of the horseshoe crab (*Limulus polyphemus*). LAL interacts with endotoxins, leading to a specific color change. The intensity of the colour change is directly proportional to the amount of endotoxin present in the sample (14). The duration of incubation during the LAL test is crucial for obtaining accurate and reliable results (15). The incubation time is carefully optimised to allow sufficient interaction between the LAL enzyme and the endotoxins in the sample. If the incubation time is not followed precisely, it can affect the sensitivity and accuracy of the assay. Insufficient incubation time may result in an incomplete reaction, leading to underestimation of endotoxin levels, while excessive incubation time can lead to non-specific reactions and false positive results.

Testing for interfering substances is an important step in the LAL test to avoid false negative results and ensure accurate detection and quantification of endotoxins (16). Interfering substances are components present in the sample matrix that can potentially interfere with the LAL assay and affect the detection of endotoxins. These substances include certain chemicals, proteins, surfactants or other compounds that inhibit or enhance the LAL reaction, leading to inaccurate results (17). To mitigate the interference caused by these substances, it is recommended to test for interfering substances. This involves spiking the sample with a known concentration of endotoxins and analysing the recovery of the spiked endotoxins in the presence of the sample matrix (7). The recovery should fall within an acceptable range (± 25% of the known spiked concentration), indicating that the sample matrix does not significantly interfere with the LAL reaction (7). If interfering substances are found to be present and affecting the accuracy of the LAL assay, additional steps may be required, such as sample dilution or sample treatment methods to reduce the interference (7, 10). Serial dilution of the sample is a common approach to dilute the interfering substances and bring the endotoxin concentration within the linear range of the LAL assay. Hence, it is important to carefully plan and execute the dilution steps, ensuring that the dilutions are accurately prepared and properly documented. Following the recommended dilution protocols provided by the LAL test kit manufacturer or regulatory guidelines is crucial to maintain the reliability and validity of the test results.

At present, no analogous tests have been conducted for MRI contrast materials, limiting the ability to directly compare results. However, the results from the LAL assay using a surgical glove were the closest available for comparison (19). In terms of the comparison of the results, the methodological approaches of the two studies differ significantly. Takahashi et al. (19) focuses on measuring endotoxin levels on glove surfaces through serial dilution and subsequent analysis, whereas the present study emphasises the comparison of endotoxin concentrations with standard solutions and the evaluation of potential interfering substances. Both results demonstrate high linearity in their measurements: *r*^2^ = 0.9975 for (19) and *r*^2^ ≥ 0.98 for the present study, indicating reliable and accurate quantification. In terms of detection and compliance, Takahashi et al. (19) highlights specific contamination on glove surfaces with significant endotoxin levels. Conversely, the present study assures compliance with regulatory limits (below 0.5 EU/mL) and confirms the absence of interfering substances, indicating no false negative results based on the difference value being above or below the spiked known endotoxin amount.

The test methods used in the present study and the previous one are different. The present study used the LAL chromogenic test, while the previous study used the turbidimetric kinetic endotoxin-specific assay using the LAL test. Both methods are used to detect endotoxins and are recognised by the WHO International Pharmacopoeia (7) but they operate based on different principles and measurement techniques. The chromogenic test used in the present study can be more straightforward but requires precise timing and careful handling of reagents, making it highly sensitive and specific for endotoxins. Meanwhile, the turbidimetric test involves continuous monitoring and may require more specialised equipment. The choice between them may depend on the sample type, required sensitivity and available instrumentation.

The US Food and Drug Administration (USFDA), Guidance for industry (18) set the maximum permissible endotoxin level for medical devices at 0.5 EU/mL or 20 EU/mL device for products that directly or indirectly contact the cardiovascular system and lymphatic system, which also including MRI contrast media. Some study in detecting pyrogen in sterile glove also referring to similar guideline (19). The pharmaceutical sector is one of the most important uses of the LAL test, especially for pyrogen detection. Because endotoxins are particularly prevalent pyrogens in pharmaceutical products, International Pharmacopeial recommendations have replaced rabbit pyrogen tests with the LAL test (20). Additionally, pyrogen testing is important for medical devices as well such as MRI contrast material. Regulatory agencies like the European Medicine Agency in Europe and the USFDA (21) must grant pre-market approval before marketing such products to manufacturers both domestically and globally, including in the US and Europe considering MRI contrast material using injectable solutions like ferum oxides. Therefore, marketing permission applications for pharmaceuticals and/or medical devices must include extensive safety data (22). However, since there is no standard value for the endotoxin limit specifically for sterile gloves and MRI contrast materials, it will be necessary to establish this value in the near future to ensure consistent safety standards.

## Conclusion

The findings from this study lead to the conclusion that the evaluated sample, a distinctive synthetic material comprising dry iron oxide nanoparticles, is improbable to possess pyrogenic qualities or trigger pyrogenic responses. This is substantiated by the demonstrated endotoxin activity falling below the stipulated regulatory threshold of 0.5 EU/mL, firmly establishing its non-pyrogenic characteristics. Furthermore, it is noteworthy that the chromogenic LAL test effectively gauged the endotoxin content in medical devices, notably in the case of MRI contrast agents.

### Future Perspective

In the future, further research can focus on developing more advanced and robust techniques for evaluating the pyrogenicity of contrast agents and other medical materials. This may involve the exploration of alternative methods to the LAL test, including recombinant technologies or other novel assays that provide enhanced specificity and sensitivity. Moreover, developing standardised protocols and guidelines for incubation times in the LAL test, specific to different sample types and endotoxin levels, would further enhance the accuracy and reliability of the assay. Automation and technological advancements in the LAL test process can also be explored to streamline the procedure, reduce human error and improve overall efficiency.

## Figures and Tables

**Table 1:** The conversion of the OD to EU/mL based the standard curve’s linear equation

Sample	Average	Absorbance (Δ)	Concentration (EU/mL)
TI	0.110	−0.020	0.026
TI SP	0.104	−0.026	0.025
ISDL 1	0.120	−0.010	0.028
ISDL SP 1	0.150	0.020	0.036
ISDL 2	0.132	0.002	0.031
ISDL SP 2	0.147	0.017	0.035
ISDL 3	0.134	0.004	0.032
ISDL SP 3	0.136	0.006	0.032
Blank	0.130	0.000	0.031

Note: ISDL = interfering substances diluted; ISDL SP = interfering substances diluted spiked; TI = test item; TI SP = test item spiked

**Table 2:** The presence of inhibitory substances based on the unspiked and spiked sample

Test item dilution	Observed spiked test item concentration	Observed unspiked test item concentration	Δ	Results
1:05	0.036	0.028	0.008	Non-inhibitory

## References

[1] Zhou IY, Catalano OA, Caravan P (2020). Advances in functional and molecular MRI technologies in chronic liver diseases. J Hepatol.

[2] Cleveland D, Long SE, Sander LC, Davis WC, Murphy KE, Case RJ (2010). Chromatographic methods for the quantification of free and chelated gadolinium species in MRI contrast agent formulations. Anal Bioanal Chem.

[3] Mukhopadhayay M, Chatterjee A (2023). Sterilization of biomaterials and medical devices with supercritical CO_2_. Sterilization and preservation: applications of supercritical carbon dioxide.

[4] Farhana A, Khan YS (2022). Biochemistry lipopolysaccharide [Internet]. StatPearls.

[5] El-Radhi AS, El-Radhi AS (2018). Pathogenesis of fever. Clinical manual of fever in children.

[6] Moorman WD (2020). Exploratory Studies into the Therapeutic and Diagnostic Capability of Blood from the Horseshoe Crab, Limulus polyphemus [PhD’s thesis].

[7] The International Pharmacopoeia (2019). 3,4 Test for bacterial endotoxins.

[8] Tamura H, Reich J, Nagaoka I (2021). Outstanding contributions of LAL technology to pharmaceutical and medical science: review of methods, progress, challenges, and future perspectives in early detection and management of bacterial infections and invasive fungal diseases. Biomedicines.

[9] Griffiths GL, Vasquez C, Escorcia F, Clanton J, Lindenberg L, Mena E (2022). Translating a radiolabeled imaging agent to the clinic. Adv Drug Deliv Rev.

[b10-20mjms3105_bc] 10ISO10993-11 Biological evaluation of medical devices, Part 11 tests for systemic toxicity, the *Limulus* amebocyte lysate (LAL).

[b11-20mjms3105_bc] 11Pierce^TM^ Chromogenic Endotoxin Quant Kit (Cat. No. A39552S) user guide.

[12] Yan KC, Sedgwick AC, Zang Y, Chen GR, He XP, Li J (2019). Sensors, imaging agents, and theranostics to help understand and treat reactive oxygen species related diseases. Small Methods.

[13] Hingorani DV, Bernstein AS, Pagel MD (2015). A review of responsive MRI contrast agents: 2005–2014. Contrast Media Mol Imaging.

[14] Li Y, Italiani P, Casals E, Tran N, Puntes VF, Boraschi D (2015). Optimising the use of commercial LAL assays for the analysis of endotoxin contamination in metal colloids and metal oxide nanoparticles. Nanotoxicology.

[15] Ostronoff CS, Lourenço FR (2015). Measurement uncertainty of chromogenic LAL assays: reaction time and proportion of endotoxin and LAL reagent affect release of p-nitroaniline. J AOAC Int.

[16] Neun BW, Dobrovolskaia MA (2018). Considerations and some practical solutions to overcome nanoparticle interference with LAL assays and to avoid endotoxin contamination in nanoformulations. Methods Mol Biol.

[17] Sheraba NS, Diab MR, Yassin AS, Amin MA, Zedan HH (2019). A validation study of the *Limulus* amebocyte lysate test as an end-product endotoxin test for polyvalent horse snake antivenom. PDA J Pharm Sci Technol.

[18] US Food and Drug Administration (FDA) (2012). Guidance for industry: pyrogen and endotoxins testing: questions and answers.

[19] Takahashi G, Kan S, Hoshikawa K, Sato K, Fujita Y, Inada K (2020). Endotoxin contamination of single-use sterile surgical gloves. Future Microbiol.

[20] Mehmood Y (2019). What is *Limulus* amebocyte lysate (LAL) and its applicability in endotoxin quantification of pharma products. Growing and handling of bacterial cultures.

[21] European Medicine Agency European Medicine Agency in Europe and the Food and Drug Administration in the USA.

[22] Tobin JJ, Walsh G (2023). Medical product regulatory affairs: pharmaceuticals, diagnostics, medical devices.

